# Leveraging mHealth Technologies for Public Health

**DOI:** 10.2196/49719

**Published:** 2024-09-12

**Authors:** Pedro Elkind Velmovitsky, Merna Kirolos, Paulo Alencar, Scott Leatherdale, Donald Cowan, Plinio Pelegrini Morita

**Affiliations:** 1 School of Public Health Sciences University of Waterloo Waterloo, ON Canada; 2 David R Cheriton School of Computer Science University of Waterloo Waterloo, ON Canada; 3 Research Institute for Aging University of Waterloo Waterloo, ON Canada; 4 Department of Systems Design Engineering University of Waterloo Waterloo, ON Canada; 5 Institute of Health Policy, Management, and Evaluation University of Toronto Toronto, ON Canada; 6 Centre for Digital Therapeutics Techna Institute University Health Network Toronto, ON Canada

**Keywords:** mobile health, mHealth, smart technology, wearables, public health, population health, apps, surveys, self-report, surveillance, digital public health, mobile phone

## Abstract

Traditional public health surveillance efforts are generally based on self-reported data. Although well validated, these methods may nevertheless be subjected to limitations such as biases, delays, and costs or logistical challenges. An alternative is the use of smart technologies (eg, smartphones and smartwatches) to complement self-report indicators. Having embedded sensors that provide zero-effort, passive, and continuous monitoring of health variables, these devices generate data that could be leveraged for cases in which the data are related to the same self-report metric of interest. However, some challenges must be considered when discussing the use of mobile health technologies for public health to ensure digital health equity, privacy, and best practices. This paper provides, through a review of major Canadian surveys and mobile health studies, an overview of research involving mobile data for public health, including a mapping of variables currently collected by public health surveys that could be complemented with self-report, challenges to technology adoption, and considerations on digital health equity, with a specific focus on the Canadian context. Population characteristics from major smart technology brands—Apple, Fitbit, and Samsung—and demographic barriers to the use of technology are provided. We conclude with public health implications and present our view that public health agencies and researchers should leverage mobile health data while being mindful of the current barriers and limitations to device use and access. In this manner, data ecosystems that leverage personal smart devices for public health can be put in place as appropriate, as we move toward a future in which barriers to technology adoption are decreasing.

## Introduction

### Background

Public health surveillance is the collection, analysis, and dissemination of data to improve population health [1-3]. These data types are the most important source of information to support decision-making and interventions by public health agencies. One of the main sources of data are surveys [4,5]. However, self-reported survey data may have substantial limitations related to self-report, including social [6-10] and recall biases [4,5,9-14]. These challenges can produce misleading results: for example, Canadian self-reported BMI data were significantly lower than BMI measured directly in a representative sample of adults [15], which can be explained by biases and limitations in self-report [15,16]. Other potential limitations include delays between collection and reporting [17,18] and costs or logistics [9,18].

In this context, an alternative is the use of mobile, wearable, and Internet of Things technologies, such as smartphones, smartwatches, and wireless scales, as additional or complementary survey and assessment tools [16,19,20] to mitigate some of these challenges, as evidenced by a recent study that used surveys and Apple Watch data to study heart rate changes in patients with COVID-19 [21].

Smart technologies have had an amazing adoption rate, with 32 million Canadians owning a smartphone [22] and almost 4 million Canadians owning a fitness wearable device [23]. Notably, smart technologies have sensors that provide zero-effort monitoring of vital signs, environmental variables, and behavioral metrics, such as heart rate, sleep, and blood pressure, among others [24]. For instance, Apple Health (AH; Apple, Inc) [25,26], one of the most popular sources of health data from sensors, collects information from smart devices that can be connected to Apple operating systems, such as smartwatches, wireless blood pressure cuffs, wireless scales, and sleep tracking mats, among others. Sensors manufactured by Apple as well as by different manufacturers can integrate with AH and read and write data to and from it. In this manner, a diverse environment of sensors can be integrated with Apple’s data repository.

These data are typically very large and can often be accessed at relatively low costs. Furthermore, the data can be composed of individuals who traditionally would not participate in health studies. Sensor data are also collected continuously, providing richer and more representative objective information that could potentially be used to complement traditional public health self-reporting and reveal new insights into the behavior of individuals in real-life environments [27].

Velmovitsky et al [28] provide an example with the Canadian Health Measures Survey (CHMS), a major Canadian public health survey consisting of an interview with the respondent, a visit to a clinic for examinations and physical measures, and the use of an activity monitor for a week. While not a traditional surveillance program, the CHMS and similar surveys provide self-reported indicators of interest for public health agencies and, therefore, can be used to illustrate the potential of mobile health data to complement traditional self-report. Indeed, several of the CHMS measures, both taken at the clinic and self-reported, could be complemented with data from smart technologies, such as body composition, heart rate, sleep behavior, and physical activity. In addition to providing additional information, these data could potentially minimize the aforementioned limitations of biases, costs, and delays. Furthermore, using data that are passively and continuously collected by personal devices for long periods can provide more accurate and representative data than the weekly fitness tracker [27,28].

### Objectives

However, there are still challenges that need to be overcome if smart, personal devices are to be used for public health, including technological, ethical, and societal challenges. One of the tenets of public health is equity [29]. In the context of smart technologies, “digital health” equity is achieved when individuals have equal opportunity to “benefit from the knowledge and practices related to the development and use of digital technologies to improve health” [30]. Digital health equity can be compromised as not everyone has equal and fair access to technology, along several dimensions (eg, income, location, and population). Other potential challenges include security, privacy, and data ownership issues as well as technological limitations (eg, lack of interoperability), which affect the integration of smart devices and public health.

These challenges and limitations must be clearly stated and recognized for public health entities to understand the potential pitfalls of using smart technologies in surveillance. It is our view that by identifying and being mindful of these, it is possible to plan accordingly and integrate new tools and technologies within public health efforts, moving toward a scenario in which these issues are mitigated and smart technologies are used to complement data collection.

This study aims to explore the potential and limitations of using smart devices in public health. It argues that these tools can enhance traditional surveillance methods, but their implementation requires careful consideration. This paper will particularly focus on the Canadian context, examining technology adoption barriers, access, and equity. It will consider the characteristics of populations that widely use smart devices as well as those that do not use or have access to these tools. In addition, a mapping of variables collected in major Canadian surveys that could potentially be gathered with AH is provided to support our view.

## Methods

This paper provides an overview of the Canadian public health data collection context and challenges, with a focus on mobile health. To support our view, we conducted a review of 3 major Canadian surveys, the CHMS; Canadian Community Housing Survey; and Physical Activity, Sedentary Behaviour and Sleep indicators, noting the data points collected by each survey. Then, we mapped the data collected by AH at the time of writing (by examining the AH app in an iPhone [Apple, Inc] device) to the survey variables.

We also reviewed the literature pertaining to mobile health research and public health, both peer-reviewed and gray literature, to further inform the snapshot of the Canadian public and mobile health context. Regarding gray literature, we looked at reports detailing the major wearable vendors as well as information on their use (eg, from the government of Canada).

Finally, we summarize these results and discuss our view on the use and adoption of smart devices in public health. Tables are included in Multimedia Appendix 1.

## Results

### AH and Canadian Surveys

Several companies now produce devices capable of capturing data in line with health metrics traditionally found in public health data collection efforts. To highlight the existing overlap, we compiled the variables that can be collected with AH (iOS 15.1) and presented them in Table S1 in Multimedia Appendix 1 [25,26]. We then compared these to the ones currently captured by 3 Canadian public health surveys. These were chosen as they are major health surveys, some of which use data from other surveys to complement indicators and, as such, provide a good overview of how the Canadian public health data collection efforts resemble data from AH.

Here, we present a summary of which AH data could supplement the CHMS (Table S2 in Multimedia Appendix 1); Canadian Community Housing Survey (Table S3 in Multimedia Appendix 1); and Physical Activity, Sedentary Behaviour and Sleep indicators (Tables S4 and S5 in Multimedia Appendix 1 for adults and children, respectively) [31,32]. For these analyses, we used the most recently completed survey cycle. Where self-report metrics are composed of many questions, we included examples of these questions and indicated which AH variables could potentially be used to complement the metrics. It should be noted that this is a snapshot of the Canadian public health surveillance system at the time of the analysis; while we reviewed each of the data points collected in the surveys and compared them to AH, the goal was not to systematically review these surveys.

As can be seen in Tables S1-S5 in Multimedia Appendix 1, several metrics, such as information on activity, symptoms, sleep, and biological characteristics (eg, height and weight), among others, can be objectively complemented by AH. These data may also provide more granular and detailed information, complementing traditional public health initiatives based on self-report with more objective data that can be used to gain further insight into the health of populations.

However, it is important to note that—as shown in the following sections—there are many challenges involved in the use of AH and smart technologies for public health, and those need to be considered and addressed to ensure digital health equity.

### Application of Mobile Health Technology in Public Health Surveillance

#### Overview

The previous section showed how many data types that are currently collected by major Canadian surveys can be complemented using objective AH data. Before discussing additional dimensions and challenges of using smart technologies for public health in the following sections, this section describes examples of health studies that successfully applied mobile device data for health research. These studies also illustrated difficulties in using smart devices. It is important to note that, while studies such as the ones by Ma et al [33] and Velmovitsky et al [34] did not focus on surveillance efforts per se as the final goal, they highlighted how mobile and wearable devices can potentially be used to collect data and gain insights into the health of individuals and study the prevalence of conditions in a population.

#### Current Efforts in Mobile and Public Health

An interesting study evaluated the levels of physical activity from players of the popular Pokémon Go mobile app using data from AH and found that the game is associated with short-term physical activity increase, particularly among more sedentary individuals [33]. To collect the data, participants were asked to take screenshots of their AH screen. It is important to note that data can be directly accessed from AH using the HealthKit API, which allows third parties to access—with user consent given in the device—the health data stored in users’ AH app, providing powerful tools for researchers to optimize data collection [25,26].

Furthermore, to make it easier for researchers to conduct studies using mobile technology, Apple has introduced the ResearchKit framework enabling the creation of visual consent flows, customizable surveys, and active tasks [26]. An example is the mPower app, developed with ResearchKit, which collects iPhone gyroscope data to better understand Parkinson disease. Initial results included approximately 10,000 enrolled participants, providing a continuous flow of data from several individuals that consented to their data being used by health researchers around the world [19]. To use ResearchKit, however, research teams should also have knowledge of mobile technology development. This leads to a challenge and opportunity, in that conducting health informatics research needs to involve a multidisciplinary research team with collaboration between the fields of public health and computer science.

Hicks et al [27] described several large-scale observational studies that use commercial mobile and wearable devices, including a study by the authors themselves which used data from >700,000 activity-tracking app users in 100 countries. This study concluded that inequality in the physical activity levels between different countries is a stronger predictor of obesity than activity levels in the country. The authors pointed out that novel sources of data from consumer apps allow researchers to gain new insights into the health and behaviors of individuals. This can be enhanced by linking mobile data with other sources, such as administrative data sets. In addition, the approach of using smart technologies, including leveraging data from pre-existing devices, allows the collection of larger observational data sets than were previously thought possible and could even be used to identify natural experiments in a population.

Furthermore, even if a population is not well represented in a data set, if the data are large enough, there could still potentially be a statistically significant number of participants that follow population distributions and allow for methodologically sound analyses. However, the authors were quick to point out the challenges with this approach, such as inaccuracy of sensors and missing data. Inequities in technology access may also lead to selection bias as individuals that use the technology or app may not be representative of the general population.

Due to its ability to generate large data sets, mobile research can also be used in conjunction with artificial intelligence methods, such as machine learning (ML), which learns patterns in data to make predictions. Indeed, ML predictive models work best with large data sets, which can be collected through smart technologies. As an example, several studies used ML to forecast COVID-19 incidence, using data from sources such as Google’s mobility data set [35]. Applying ML methods will also require further multidisciplinary knowledge in computer and data science.

Several efforts are also underway to create ecosystems that allow users to register their devices and continuously donate data for research. For example, the ecobee company, producer of a smart thermostat device, has launched the Donate Your Data program [36,37], which allows thermostat owners to anonymously share their data with researchers. The Ubiquitous Health Technology Lab has conducted studies using this data set [38-40] and has recently deployed a web platform that allows individuals to access study information and enroll their personal Fitbit (Google Fitbit) and Ecobee (Ecobee Inc) devices. Once enrolled, data from the devices are collected by the Ubiquitous Health Technology Lab once a day [41].

Recently, Velmovitsky et al [34] developed a mobile platform that collects Apple Watch electrocardiogram data through HealthKit to predict stress levels using ML. By quantifying stress levels, public health agencies could potentially apply interventions such as notifying users or asking if they would like to open a meditation application. Notably, this study gave devices to participants rather than use data already collected from their personal devices; therefore, the data set used was not particularly large and did not represent surveillance but rather worked as a pilot study to illustrate the benefits of mobile and wearable applications to public health. Preliminary results of the study achieved model accuracies (ie, trained using the entire data set according to several demographic factors) of approximately 55% to 60%, consistent with the low end of state of the art for ML stress prediction models using real-life data. The models had high specificity, accurately identifying when an individual is not stressed, but were less successful in predicting when an individual was stressed. The process of collecting HealthKit data in this study and the mobile app are described elsewhere [42,43]. Interestingly, much like the previous work by Hirten et al [21], this study also found the SD of interbeat interval of normal sinus beats to be one of the most important features, in this case for predicting stress.

#### Skilled Workforce

In this context, one of the challenges with mobile health research, as pointed out by Hicks et al [27], is that researchers typically need multidisciplinary experience in computer science and health research to use these tools to their full potential in their studies. If one wishes to use the HealthKit API, for example, it would be necessary to program a data collection script that uses Apple’s programming language, Swift. In other words, researchers looking to use mobile and wearable data need to have knowledge in at least 2 disparate fields—health care (to design proper studies and analyze and interpret the data) and computer science (for data collection with mobile devices)—and having such multidisciplinary knowledge may be challenging.

#### Data Management and Governance

**Figure 1:**
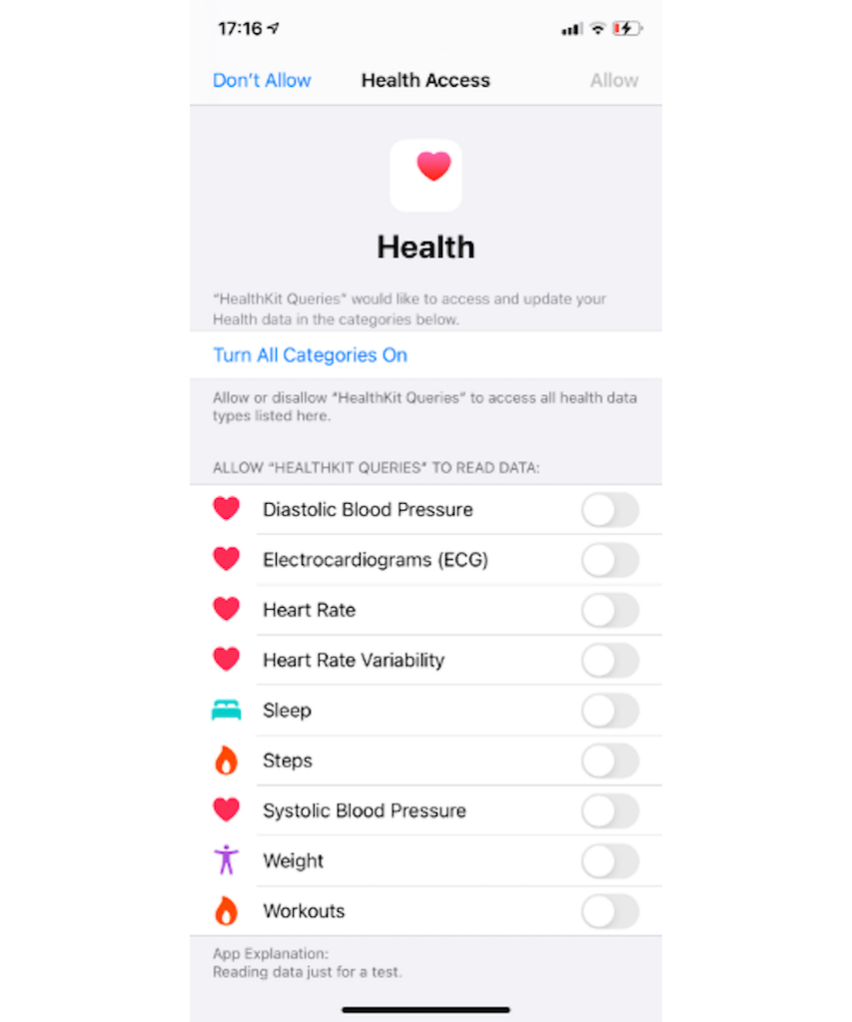
HealthKit consent screen.

In case computer science expertise is lacking, public health researchers may be required to find more creative ways to collect the data, as shown in Figure 1 with the screenshot requested of users. Another path available to collect the data without the need for coding is to export the data directly through the AH app in the XML format, although that may still require coding skills to handle the data inside the file. Missing data might be a particular problem for studies dealing with real-life data collection, with a lot of factors outside the researcher’s control (eg, errors in measurement due to movement or caused by individuals forgetting to wear the device or not wearing it correctly) [34,44]. In this case, careful data processing is necessary, which may involve using data imputation algorithms or removing the missing intervals [44].

Velmovitsky et al [28] discussed the role of big data in precision medicine and public health. In particular, an overview of different big data types is provided, which include omics, clinical, social data (ie, social media data); patient-generated health data (ie, data from personal smart devices); and environmental and demographic data. Among challenges related to mobile health research, difficulty in linking patient-generated health data with clinical data is highlighted as a lot of medical and administrative information may be siloed in providers’ systems, which are not typically interoperable and cannot be integrated, in addition to security and privacy issues. The authors also suggest areas that could be improved with the use of big data, such as disease surveillance. A recent example of the benefits of mobile devices for this field is the previously mentioned observational study that used surveys and Apple Watch data to identify patients with COVID-19 [21]. The SD of the interbeat interval of normal sinus beats, a heart rate variability metric, differed significantly in the 7 days before and after COVID-19 diagnosis compared to uninfected periods, suggesting that the Apple Watch could potentially be used as a predictive tool for COVID-19.

#### Accuracy of Mobile Devices

There have also been several studies that compared the accuracy of mobile devices to gold standard measurements. Hart et al [45] found that the activPal Professional device and the Bouchard Activity Record (ie, a self-report log that assesses time spent sitting, lying, standing, and in physical activity) showed moderate to high agreement and correlation for total and concurrent time spent walking and in sedentary behavior. However, it is not always the case that the results are successful. A study comparing the ActiGraph device with the International Physical Activity Questionnaire found low to moderate correlations with International Physical Activity Questionnaire overestimating sitting and vigorous activity, for instance [46]. In fact, a systematic review of wearables found that data may be underestimated or overestimated in several devices and models [47], and a study found that the Fitbit Flex differed from the ActiGraph GT3X+ in reporting steps in free-living conditions (ie, differences increased with the number of steps taken) [48].

Regarding the Apple Watch, the heart’s RR intervals measured with the device during relaxation and stress states were shown to have high reliability and agreement with signals obtained from the Polar H7 chest strap [49], suggesting that heart data from Apple Watch are accurate. There is also limited but promising evidence on the accuracy of Apple Watch sleep data [50]. Finally, it is important to note that there is growing evidence of inaccuracies in the use of photoplethysmography green light signaling in many wearables for individuals with darker skin tones compared to those with lighter skin tones, which may introduce biases in the analyses [51].

It is often challenging to compare the accuracy of mobiles and wearables as studies tend to use different metrics for assessing validity and reliability, making the comparison between devices difficult [47]. In addition, the speed at which new device models are released, or systems updated, causes studies to quickly become obsolete, especially if there are significant differences between the sensors and algorithms used to measure data [16,48]. Differences in models may limit the applicability of mobiles and wearables in population-level studies over time, as it is not possible to guarantee comparability, and that remains a significant issue [16,48]. To date, the literature suggests that several mobile health devices and metrics are in line with gold standard measurements in public health. However, some devices continue to fall below the standard, and further development will be required before they can be implemented in public health.

### Technology Adoption: Facts and Challenges

#### Overview

In this section, we present results relating to the adoption of smart technologies. As of 2022, Apple, Samsung, and Fitbit mainly compose the Canadian wearable market [52]. A summary of the characteristics of each company is shown in Table S6 in Multimedia Appendix 1.

Garmin and Samsung have a similar market share (13%), but since Garmin is more focused on athleticism, we restrained our analyses to Samsung due to its focus on smart devices. With a focus on the Canadian context, we also describe major challenges to technology adoption. While the implications for public health are presented in the Discussion section, it should be clear from the discussion that follows that there is no brand that acts as a “silver bullet” that encompasses the entire population. In this manner, by choosing one company over another as a focus of a study (eg, allowing Apple Watch users to bring their own devices) would exclude part of the population. Therefore, it is essential that researchers take into account who is using each of the devices and ensure they understand who is being included or excluded from the study.

#### Characteristics of Major Mobile and Wearable Companies

##### Apple

As can be seen in Table S6 in Multimedia Appendix 1, Apple has the largest wearable market share in Canada (41%) [52]. Compared to other brands, Apple has the largest share of users aged between 18 and 29 years (35%), with no significant difference between the percentage of female and male users [52]. In contrast, at approximately 15%, Apple has the lowest share of users aged between 50 and 64 years old compared to other brands [52]. Apple users are typically more educated, and 50% of Apple users have a high monthly income [52]. The type of community that Apple users are in also differs from that of other companies, with the majority living in larger cities. Of note, 67% of Apple users reported accessing the internet through their smartwatches compared to other wearable users (53%) [52]. This could be related to Apple users having a higher income, which allows them to obtain more devices with internet access (as further detailed in Income subsection). Apple’s popularity has grown, with Apple wearable users increasing by 11% in the past 2 years [52].

On a global scale, Apple’s geographic segment is primarily in the United States and urban cities [53]. The company’s marketing strategy is aimed at consumers with high purchasing power and career focus, such as those in professional executive positions. Furthermore, Apple relies on the loyalty of its customers who typically continue to purchase all their electronics from Apple. Apple’s brand value was approximately US $947 billion, due in large part to customer loyalty and brand recognition as an exclusive and luxury product [54].

##### Fitbit

Fitbit produces the second most used wearable in Canada, with a market share of 38% [55]. In Canada, most Fitbit users are female, and Fitbit has the highest share of users aged between 50 and 64 years [55]. In contrast, Fitbit has the lowest share of users aged between 18 and 29 years. In total, 42% of Fitbit users have a high monthly income and most live in larger cities [55]. A large share of Fitbit users are educated. Of note, Fitbit users were found to access the internet less often through their devices (46%) compared to the average wearable user (53%) [55]. Furthermore, Fitbit users performed more hiking activities in comparison to any other wearable users. They also engaged more in aerobic and cardio physical activities compared to other wearable users, suggesting that Fitbit users are in general more interested in fitness and exercising [55].

On the global scale, as of 2021, Fitbit has sold >127 million wearables worldwide with 111 million registered users [56]. For reference, Apple has the highest share of the wearable device market with 160 million sold globally. Unlike Apple, Fitbit’s wearable market share has declined by 12% in the past 2 years [55]. Fitbit was valued at US $2.1 billion when Alphabet Inc purchased the company in 2021 [56].

##### Samsung

Samsung produces the third most used wearables in Canada with a market share of 13% [57]. Compared to other brands, Samsung has the highest share of users aged between 30 and 39 years (30%), and most users are male (57%). In addition, 47% of the users have a high monthly income and 21% have a master’s or doctoral degree. Most Samsung users also live in larger cities in Canada [57].

On a global scale, Samsung’s geographical segment is primarily in the Asian market sector and urban cities. Globally, Samsung’s main users are adults, and their products are marketed toward society in general. Samsung has products that are for users with both low and high purchasing power, expanding the brand’s target market [53].

#### Barriers and Enablers for Population Uptake of Mobile Devices

##### Security, Privacy, and Data Ownership Issues

Security and privacy issues must be addressed during health data collection and are particularly important challenges to the collection, storage, and use of data from smart technologies.

In Canada, the Personal Information Protection and Electronic Documents Act (PIPEDA) regulates the collection, use, and disclosure of personally identifiable information (PII) for private-sector organizations involved in a commercial activity. This includes pharmacies, providers, and laboratories [58]. This federal act applies to all types of PII [59,60]. Several provinces have adopted health sector laws dealing with personal health information, some of which are deemed substantially similar to PIPEDA and taking precedence in these provinces (Table S7 in Multimedia Appendix 1) [59-61]. PIPEDA still applies when personal health information is transferred provincially or nationally.

PIPEDA is based on 10 principles (Table S8 in Multimedia Appendix 1) [60,62]. The principle of safeguards mandates that PII “be protected by security safeguards appropriate to the sensitivity of the information” [60]. Provincial health care acts define similar protective measures; for example, the Personal Health Information Protection Act states that health information custodians must “take steps that are reasonable in the circumstances to ensure that personal health information...is protected against theft, loss and unauthorized use or disclosure...” [58,62]. To inform health custodians, the Information and Privacy Commissioner of Ontario listed recommended safeguards (Table S9 in Multimedia Appendix 1) [63]. In other words, Canadian privacy laws require that health custodians protect PII by appropriate measures. What constitutes an appropriate measure will depend on the sensitivity of the information and the custodian’s circumstances, including the type or size of the organization and if the data are shared with third parties [59,63]. Organizations must obtain informed consent for the collection, use, and disclosure of PII and state their purposes for data collection (Table S8 in Multimedia Appendix 1).

Different countries and regions have different regulations. The Health Insurance Portability and Accountability Act, which applies to subsets of health custodians in the United States, offers a similar but more comprehensive list of technical, physical, and administrative safeguards [64], while the General Data Protection Regulation regulates the handling of PII in the European Union and is considered one of the most comprehensive privacy legislation in the world. General Data Protection Regulation and Health Insurance Portability and Accountability Act guidelines can also help Canadian health custodians to understand their security needs and implement adequate safeguards.

The issue of security and privacy is further complicated when ownership of the data is considered. In other words, are the data owned by the individuals who generated the data, the corporations that manufactured the data collection devices, or other stakeholders? While a comprehensive discussion of data governance is outside the scope of this paper, this legal and societal issue still needs to be addressed when discussing data collection with mobile and wearable technologies. Velmovitsky et al [65] highlighted potential trust issues in the data collection process between corporations, third-party solutions, individuals, providers, and regulations. In particular, individuals using such technologies need to trust that the corporations (eg, Apple, Samsung, and Fitbit) and research and personal applications (eg, fitness and research apps) are using the data only for the purposes originally consented to. Regulatory agencies need to ensure that regulations (such as the ones discussed in the previous paragraph) are being respected by these entities.

Micheli et al [66] further highlighted asymmetries of power regarding technology corporations having large and unrestricted access to data, which could result in privacy violations such as the case of a Facebook data leak, which enabled Cambridge Analytica to use these data improperly for voter profiling [67]. The authors further highlighted governance models proposed in the literature, including (1) data sharing pools in which data are digitally shared between partners, with contracts stipulating the conditions of use; (2) data cooperatives, which are similar to sharing pools but with more involvement of data participants, which have more control over the data sharing process; (3) public data trusts, which involve a public entity accessing citizen and company data; and (4) personal data sovereignty, in which data participants have complete control over their data and sharing permissions. The open issue of data ownership is particularly important in the context of research and public health surveillance, as the gateways offered by companies such as the HealthKit API are controlled by these entities, and as such, access to data could potentially be charged in case mobile health data are increasingly used for research.

Researchers who collect, use, and disclose PII for noncommercial activities are not typically subject to PIPEDA but must still get approval from the appropriate review ethics boards, which typically also require safeguards according to the sensitivity of the data [58,59]. Furthermore, public health agencies are generally not subject to PIPEDA but to federal, provincial, and territorial laws dealing with PII in their region [68]. For example, the Public Health Agency of Canada is subjected to the federal Privacy Act, which delineates individual privacy rights in relation to the federal government [68].

It should also be noted that applications that allow data sharing between smart technologies typically have their consent mechanisms. For example, the HealthKit API requires that individuals first give consent to each data type for this data collection [25], as shown in Figure 1. In addition to these mechanisms, researchers and public health agencies should still obtain consent for data collection following applicable regulations.

In summary, any third-party entity collecting health data for commercial purposes (eg, private health care providers and mobile app developers) are subject to PIPEDA and must respect the principles to protect personal information. Researchers and public health agencies are subjected to their own ethics boards and privacy regulations, which typically also require obtaining consent for data collection and use.

##### Internet Access by Canadians

#### Overview

As the use of mobile and wearable data in health continues to grow, researchers must acknowledge and address inequalities in technology access. Disparities may lead to selection bias as individuals that use the technology or app may not be representative of the general population.

In 2020, nearly 6% of Canadians did not have internet access at home. Of these, approximately 63% felt no need for it, 26% found the service costs to be too high, and 13% found the equipment costs prohibitive [69]. From 2015 to 2023, Canada’s internet users have steadily increased, reaching 36 million. In other words, approximately 94% of Canada’s population has access to the internet [70]. In this manner, while internet access remains a barrier for some of the population, most Canadians currently have access to the internet, and this number is projected to increase.

The standards set by the Canadian Radio-television and Telecommunications Commission (CRTC) for internet connectivity are a minimum download speed of 50 mbps and an upload speed of 10 mbps. An internet speed of >50 mbps allows Canadians to perform multiple web activities and have various devices connected to the internet at once [69]. Approximately 72% of Canadian households have achieved the CRTC standards for internet connectivity. Regarding mobile data, 80% of Canadians reported having a personal mobile data plan, with only 1.5% reporting a mobile data plan without internet connection [69]. Therefore, without good internet speed and connection, the use of mobile and wearable devices for data collection is severely limited. The government of Canada has set a goal of having 98% of Canadians with access to high-speed internet by 2026 and 100% of Canadians by 2030 [71].

In this manner, internet access is a major factor in smart technology adoption. Many barriers limit the use of technology and high-speed internet for Canadians, such as household income, age, geographic location, and ethnicity. Understanding these barriers to adoption is important when using mobile health data to address health inequalities, and they will be expanded in the next sections.

#### Income

The most prevalent barrier to internet access for Canadians is low household income. The digital equity report by Deloitte found that, among the survey participants who did not have a data plan, 68% reported high costs as a barrier [72]. In addition, household income can alter an individual’s perception of technology and digital services: people earning >CAD $150,000 annually were likelier to agree (74%) that internet and new technologies had a positive impact on their lives compared to those earning <CAD $40,000 (49%) [72]. This disparity can manifest itself in differences in the quality of internet service and the range of digital tools individuals have access to.

Indeed, internet speed and household income are highly correlated. Households with lower incomes are more likely to fall below CRTC thresholds compared to households with higher incomes. In fact, most households earning <CAD $40,000 (CAD $1=US $1.3) annually do not meet the CRTC target, which is 19% higher than the national average and 28% higher than the highest income category [72]. Furthermore, families with an annual household income ≥CAD $200,000 had access to internet speeds that were approximately 30 mbps faster than those with an income of <CAD $20,000 [72]. With an additional 30 mbps, a household could connect to 3 more devices, including phones and computers. Therefore, without high-speed internet, digital health equity is severely affected, as individuals may not be able to have internet connection or access to smart technologies.

#### Urban Versus Rural Geographic Locations

Due to Canada’s vast size and dispersed population, individuals residing in rural and remote geographical locations face additional challenges in accessing high-speed internet. Rural and remote regions encounter distinct challenges concerning internet cost and speed, which can be attributed in large part to Canada’s vast size and dispersed population.

Within Canadian Census Metropolitan Areas or Census Agglomerations (CMA or CAs), 95% of households had access to a home internet connection. For households residing outside a CMA or CA, this figure drops to 88%. An even greater geographic disparity exists when one considers access to high-speed internet with download speeds of >50 mbps. Only 48% of people living outside CMA or CAs meet the CRTC target compared to 76% of respondents residing within these areas, and 73% of the people have a mobile data plan outside CMA or CAs compared to 81% residing within these areas [69].

In particular, Canadian Indigenous communities are underrepresented in the digital landscape; only 39% of the First Nation reserves in Canada met the CRTC threshold for high-speed internet [72]. Researchers must consider these geographic disparities in digital equity when implementing and collecting data from devices.

#### Older Adults

Historically, older adults have used less technology than younger populations [73]. In general, older adults typically have higher anxiety when using new technologies, and declining visual, motor, hearing, and cognitive impairments can affect technology acceptance [74]. Notably, 1 in 3 older adults aged >75 years reported frustration when using unfamiliar technologies [72].

Older adults may also not want to use any additional apps, stay limited to call and messaging functions, and decide not to have a device due to cost [74]. In the 3 brands detailed above (Characteristics of Major Mobile and Wearable Companies section), individuals aged between 50 and 64 years composed the lowest share for Apple and Samsung (15% and 14%, respectively), with a better representation in Fitbit at 32%. However, a poll conducted during the pandemic revealed that the number of Canadians aged ≥65 years who own a smartphone increased (65% in 2020 from 58% in 2019), and 83% of owners used it daily [75]. The pandemic also caused older adults to increase their technology use in general, for example, through video calls or using social media to message family and friends [75]. Indeed, in 2020, in total 72% of Canadians aged >65 years revealed that they now feel confident using technology [75], indicating that, although age could be a barrier to technology adoption, it seems that it is diminishing. This also remains true in other geographic locations, such as in the United States, where smartphone ownership and social media use among older adults is increasing, with 61% of older adults aged >65 years owning a smartphone and 45% using social media, with these figures increasing to 83% and 73% for individuals aged 50 and 64 years [73].

Although the technology access gap for older adults is becoming smaller, barriers still remain that need to be addressed to ensure equitable access, including individual (eg, physical aging, sensory impairments, and cognitive limitations) and technological barriers [76]. Indeed, one of the most frequent obstacles to accessing technology among older adults is physical aging, particularly hearing and vision impairments. Decreases in motor control, such as tremors in the hands, also make it difficult to use the devices, especially those with small screens. Lack of experience with technology, perception of their own proficiency in using the devices, and a general aversion to technology may also make adoption difficult among other adults [76]. Furthermore, technological functional barriers, such as small screen and text sizes, as well as complex functionalities that are intuitive or assume that the user has prior experience with the technology, also negatively impact adoption. The limited availability of technology devices suited or adapted to older adults also poses a challenge. Finally, the cost associated with purchasing electronic devices and data is also a significant factor limiting technology adoption; while this is true for most populations, it particularly affects older adults if they rely on a restricted or fixed income, such as government pensions [76].

#### Ethnicity

An individual’s ethnocultural background can affect technology adoption. For example, individuals of Middle Eastern, North African, and South Asian descent are more likely to view cost as a significant barrier to accessing digital technologies compared to both the national average and individuals of European descent [72].

Furthermore, racially motivated discrimination, cyberbullying, and harassment are prevalent in web spaces. Individuals of Indigenous, Middle Eastern, Asian, or African descent are likelier (60%) to have experienced online bullying or discrimination compared to individuals of White or European descent (25%) [72].

In contrast, it is also important to note that Canadians of Indigenous, Middle Eastern, Asian, or African descent use the internet as a means of connecting with others who share their ethnocultural background and finding individuals who can relate to their experiences. In Canada, 80% of Indigenous individuals used the internet to maintain regular connections with members of their community, which is significantly higher than the national average of 50% [72]. Nevertheless, digital inequity remains a significant challenge for Indigenous communities across Canada, relating to historical failures in recognizing indigenous rights, which have contributed to longstanding and wide-ranging socioeconomic disparities between Indigenous and non-Indigenous populations.

## Discussion

### Implications for Public Health

From the aforementioned sections, it is clear how data from smart technologies can be used to support health sciences and public health efforts, complementing self-report metrics with objective data and leveraging personal devices to provide continuous, passive data collection from large populations.

Indeed, studies focusing on mobile data sets are already underway. While traditional data collection methods, typically focused on self-report, have years of use and validation—as evidenced by the major surveys in use—they can potentially be complemented by objective sensor data collected passively through smart technologies that are widely adopted by Canadians and worldwide. This will allow researchers and public health specialists to access a larger volume of continuous, real-world, and real-time data for decision support and to gain new insights into the health of individuals and populations. These stakeholders should still respect the applicable security and privacy regulations, mandates from review ethics boards, and obtain user consent.

It is our view that a system that allows users to share their data with public health organizations—such as a larger version of the mobile platform suggested by Velmovitsky et al [34] and by the Ubiquitous Health Technology Lab [41]—would be beneficial in supporting health efforts, research, and interventions.

However, scientists conducting studies based on mobile health population data must be aware of the barriers and challenges identified in this paper and take them into account when designing their studies and collecting data. For example, these methods may not be appropriate for certain ethnicities, older populations, or individuals with lower income or those located in geographically distant areas. Digital health equity concerns must be addressed to ensure all populations benefit from the use of smart devices, and in case populations without equal access to mobile technologies or internet are part of the study, special care must be taken to avoid digital exclusion.

In addition, to mitigate some of these challenges, scientists can consider the characteristics of the population that uses each device. For example, if populations with lower income are the focus of a study, it would make more sense to leverage Samsung personal devices than Apple ones, as Apple products target individuals with higher purchasing power. On the same token, Fitbit devices can better target individuals with a prior interest in physical activity. Careful consideration must also be taken to ensure the devices have prior evidence suggesting that the collected data have good agreement and correlation with gold standard measurements.

Another important factor to note is that a lot of these barriers are already recognized by the government of Canada and other stakeholders, and efforts are in place to mitigate or eliminate them over the next years. The Canadian government has a goal of enabling all Canadians to access high-speed internet by 2030 [71], and older adults are becoming increasingly comfortable with technology. In a few years, it is likely that some of the barriers may not be present at all. In addition, new technologies—such as the implementation of 5G—can greatly reduce some of the challenges, for example, by increasing the number of devices that can be connected to a single point as well as the speed of data collection and transfer [77].

If researchers and public health organizations develop methods and guidelines for collecting and using personal health data now—with careful considerations on current issues regarding adoption, access, privacy, ownership, and equity—they will be more prepared to use this information in the future when those barriers are greatly diminished. For example, new standards and best practices can be created on how to obtain, process, secure, and store mobile health data; how to deal with different device models; how to obtain consent in studies using mobile data; or how to address the needs of certain populations. In addition, more studies using mobile and wearable data can generate evidence to demonstrate the importance of these devices for public health to decision makers.

The lack of interoperability between devices, which adds additional complexities, must also be considered; if a public health agency develops a system that extracts data from Fitbit devices, for instance, the same data pipeline will not work for Samsung or Apple products. Different programming languages, APIs, and protocols need to be used. This may affect potential studies as having larger and more robust data sets from a larger population would lead to more representative and quality data. The issue of interoperability must be carefully considered when designing and creating population-wide data collection systems and should also be integrated into the development of standards and best practices.

### Conclusions

In conclusion, the sooner population-wide data collection and surveillance systems using mobile technology are in place, the sooner specialists can take advantage of these data. By the same token that contact tracing apps had to be developed quickly during the COVID-19 pandemic and there was not a wide system available and in place for managing disease spread before it in most countries, it is our view that by being proactive, anticipating the need, and developing these systems in parallel to the process of eliminating barriers to device and internet access, health scientists will be better prepared to deal with the challenges of tomorrow while taking advantage of the opportunities that the future will bring.

## Appendix

Multimedia Appendix 1 Supplementary tables.

## Abbreviations

AH: Apple Health
CHMS: Canadian Health Measures Survey
CMA or CA: Census Metropolitan Areas or Census Agglomeration
CRTC: Canadian Radio-Television and Telecommunications Commission
ML: machine learning
PII: personally identifiable information
PIPEDA: Personal Information Protection and Electronic Documents Act

## Data Availability

All data generated or analyzed during this study are included in this published article (and its supplementary information files).
