# Supplementary material for: Leveraging mHealth Technologies for Public Health
Source: JMIR Public Health Surveill. 2024 Sep 12;10:e49719. doi: 10.2196/49719 (PMC11427850; doi:10.2196/49719)
Supplement: Multimedia Appendix 1 [file publichealth_v10i1e49719_app1.docx]

Table 1: Variables collected by AH, per Group

| **Group** | **Variable Name** |
| --- | --- |
| Activity | Flights Climbed |
| Activity | Steps |
| Activity | Walking + Running Distance |
| Activity | Active Energy |
| Activity | Exercise Minutes |
| Activity | Resting Energy |
| Activity | Stand Hour |
| Activity | Cardio Fitness(VO2 max ) |
| Activity | Workouts |
| Activity | Cycling Distance |
| Activity | Downhill Snow Sports Distance |
| Activity | NikeFuel |
| Activity | Pushes |
| Activity | Swimming Distance |
| Activity | Swimming Strokes |
| Activity | Wheelchair Distance |
| Activity | Stand Minutes |
| Activity | Move Minutes |
| Mindfulness | Mindful Minutes |
| Nutrition | Biotin |
| Nutrition | Caffeine |
| Nutrition | Calcium |
| Nutrition | Carbohydrates |
| Nutrition | Chloride |
| Nutrition | Chromium |
| Nutrition | Copper |
| Nutrition | Dietary Cholesterol |
| Nutrition | Dietary Energy |
| Nutrition | Dietary Sugar |
| Nutrition | Fiber |
| Nutrition | Folate |
| Nutrition | Iodine |
| Nutrition | Iron |
| Nutrition | Magnesium |
| Nutrition | Manganese |
| Nutrition | Molybdenum |
| Nutrition | Monounsaturated fat |
| Nutrition | Niacin |
| Nutrition | Pantothenic Acid |
| Nutrition | Phosphorus |
| Nutrition | Polyunsaturated Fat |
| Nutrition | Potassium |
| Nutrition | Protein |
| Nutrition | Riboflavin |
| Nutrition | Saturated Fat |
| Nutrition | Selenium |
| Nutrition | Sodium |
| Nutrition | Thiamin |
| Nutrition | Total Fat |
| Nutrition | Vitamin A |
| Nutrition | Vitamin B12 |
| Nutrition | Vitamin B6 |
| Nutrition | Vitamin C |
| Nutrition | Vitamin D |
| Nutrition | Vitamin E |
| Nutrition | Vitamin K |
| Nutrition | Water |
| Nutrition | Zinc |
| Sleep | In Bed |
| Sleep | Asleep |
| Body Measurements | Body Fat Percentage |
| Body Measurements | Body Mass Index |
| Body Measurements | Height |
| Body Measurements | Weight |
| Body Measurements | Lean Body Mass |
| Body Measurements | Waist Circumference |
| Body Measurements | Basal Body Temperature |
| Body Measurements | Body Temperature |
| Body Measurements | Electrodermal Activity |
| Heart | Heart Rate |
| Heart | Resting Heart Rate |
| Heart | Walking Heart Rate Average |
| Heart | Cardio Fitness (VO2 Max) |
| Heart | Cardio Fitness Notifications |
| Heart | Peripheral Perfusion Index |
| Heart | High Heart Rate Notifications |
| Heart | Low Heart Rate Notifications |
| Heart | Irregular Rythym Notifications |
| Heart | Heart Rate Variability (HRV) |
| Heart | Blood Pressure |
| Heart | Electrocardiogram (ECG) |
| Symptoms | Abdominal Cramps |
| Symptoms | Acne |
| Symptoms | Appetite Changes |
| Symptoms | Bladder Incontinence |
| Symptoms | Bloating |
| Symptoms | Body and Muscle Ache |
| Symptoms | Breast Pain |
| Symptoms | Chest Tightness or Pain |
| Symptoms | Chills |
| Symptoms | Congestion |
| Symptoms | Constipation |
| Symptoms | Coughing |
| Symptoms | Diarrhea |
| Symptoms | Dizziness |
| Symptoms | Dry Skin |
| Symptoms | Fainting |
| Symptoms | Fatigue |
| Symptoms | Fever |
| Symptoms | Headache |
| Symptoms | Heartburn |
| Symptoms | Hot Flashes |
| Symptoms | Loss of Smell |
| Symptoms | Loss of Taste |
| Symptoms | Lower Back Pain |
| Symptoms | Memory Lapse |
| Symptoms | Mood Changes |
| Symptoms | Nausea |
| Symptoms | Night Sweats |
| Symptoms | Pelvic Pain |
| Symptoms | Rapid, Pounding or Fluttering Heartbeat |
| Symptoms | Runny Nose |
| Symptoms | Shortness of Breath |
| Symptoms | Skipped Hearbeat |
| Symptoms | Sleep Changes |
| Symptoms | Sore Throat |
| Symptoms | Vaginal Dryness |
| Symptoms | Vomiting |
| Symptoms | Wheezing |
| Vitals | Heart Rate |
| Vitals | Blood Pressure |
| Vitals | Body Temperature |
| Vitals | Respiratory Rate |
| Vitals | Blood Glucose |
| Vitals | Menstruation |
| Vitals | Blood Oxygen |
| Cycle Tracking | Abdominal Cramps |
| Cycle Tracking | Acne |
| Cycle Tracking | Appetite Changes |
| Cycle Tracking | Basal Body Temperature |
| Cycle Tracking | Bladder Incontinence |
| Cycle Tracking | Bloating |
| Cycle Tracking | Breast Pain |
| Cycle Tracking | Cervical Mucus Quality |
| Cycle Tracking | Constipation |
| Cycle Tracking | Contraceptives |
| Cycle Tracking | Diarrhea |
| Cycle Tracking | Dry Skin |
| Cycle Tracking | Fatigue |
| Cycle Tracking | Hair Loss |
| Cycle Tracking | Headache |
| Cycle Tracking | Hot Flashes |
| Cycle Tracking | Lactation |
| Cycle Tracking | Lower Back Pain |
| Cycle Tracking | Memory Lapse |
| Cycle Tracking | Menstruation |
| Cycle Tracking | Mood Changes |
| Cycle Tracking | Nausea |
| Cycle Tracking | Night Sweats |
| Cycle Tracking | Ovulation Test Result |
| Cycle Tracking | Pelvic Pain |
| Cycle Tracking | Pregnancy |
| Cycle Tracking | Pregnancy Test Result |
| Cycle Tracking | Progesterone Test Result |
| Cycle Tracking | Sexual Activity |
| Cycle Tracking | Sleep Changes |
| Cycle Tracking | Spotting |
| Cycle Tracking | Vaginal Dryness |
| Hearing | Headphone Audio Levels |
| Hearing | Audiogram |
| Hearing | Environmental Sound Levels |
| Hearing | Noise Notifications |
| Hearing | Headphone Notifications |
| Respiratory | Cardio Fitness(VO2 Max) |
| Respiratory | Forced Expiratory Volume, 1 sec |
| Respiratory | Forced Vital Capacity |
| Respiratory | Inhaler Usage |
| Respiratory | Oxygen Saturation |
| Respiratory | Peak Expiratory Flow Rate |
| Respiratory | Respiratory Rate |
| Respiratory | Six-Minute Walk |
| Mobility | Double Support Time |
| Mobility | Step Length |
| Mobility | Walking Speed |
| Mobility | Walking Asymmetry |
| Mobility | Walking Steadiness |
| Mobility | Stair Speed: Up |
| Mobility | Stair Speed: Down |
| Mobility | Cardio Fitness (VO2 Max) |
| Mobility | Six-Minute Walk |
| Mobility | Walking Steadiness Notifications |
| Other | Alcohol Consumption |
| Other | Blood Alcohol Content |
| Other | Blood Glucose |
| Other | Handwashing |
| Other | Inhaler Usage |
| Other | Insulin Delivery |
| Other | Number of Times Fallen |
| Other | Sexual Activity |
| Other | Toothbrushing |
| Other | UV Index |
| Immunizations | COVID-19 Vaccine Records |
| Health Details | Name |
| Health Details | Date of Birth |
| Health Details | Sexual Activity |
| Health Details | Blood Type |
| Health Details | Fitzpatrick Skin Type |
| Health Details | Wheelchair Distance |
| Health Details | Medication That Affects Heart Rate |

Table 2: CHMS Measures related to Possible AH Variables

| **Type** | **Description/Question Example** | **AH** | **Possible AH Variables** |
| --- | --- | --- | --- |
| **Clinic Component** |  |  |  |
| Height | Standing height measured during clinic visit | Yes | Height |
| Weight | Weight measured during clinic visit | Yes | Weight |
| Neck Circumference | Neck circumference measured during clinic visit | N/A | N/A |
| Waist Circumference | Waist circumference measured during clinic visit | Yes | Waist cirumference |
| Resting blood pressure | Device applied in the clinic to measure resting blood pressure | Yes | Blood pressure |
| Heart Rate | Device applied in the clinic to measure heart rate | Yes | Heart Rate, Resting Heart Rate, Walking Heart Rate Average, Heart Rate Variability |
| Vision Assessment | Consists of several tests in the clinic: visual acuity, intraocular pressure, visual field, and retinal photography | N/A | N/A |
| Cardiovascular fitness | Measured using the Canadian Aerobic Fitness test, in which individuals go up and down the steps for several minutes to measure “the efficiency of lungs and heart in delivering oxygen to the exercising muscles as well as the efficiency of these exercising muscles in using the oxygen”. | Yes | Flights Climbed, Steps, Walking + Running Distance, Active Energy (kcal), Exercise Minutes, Resting Energy, VO_2_ Max, Workouts, Oxygen Saturation, Forced Expiatory Volume 1 sec, Forced Vital Capacity, Peak Expiratory Flow Rate, Respiratory Rate |
| Grip Strength | Measured in the clinic with a device called dynamometer that is squeezed as hard as the individual can. | N/A | N/A |
| Sit and Reach | Measured in the clinic, individuals sit on a mat and lean forward at the hips | N/A | N/A |
| Bone Mineral Content | X-ray at the clinic | N/A | N/A |
| Vertical jumps | Measured in the clinic with two tests: multiple two-legged hoping test, and vertical jump test | N/A | N/A |
| Level of Physical Activity | Measures the intensity, time, duration and frequency of the activity with a physical activity monitor wore for seven days following the clinic visit | Yes | Steps, Walking + Running Distance, Active Energy (kcal), Exercise Minutes, Resting Energy, Stand Hour, VO_2_ Max, Workouts, Cycling Distance, Downhill Snow Sports Distance, NikeFuel, Pushes, Swimming Distance, Swimming Strokes, Weelchair Distance, Stand Minutes |
| Blood samples | Blood samples from respondent at clinic | N/A | N/A |
| Urine samples | Urine samples from respondent provided at home | N/A | N/A |
| **Household Questionnaire** |  |  |  |
| General health (GEN) | Using a scale of 0 to 10, where 0 means "Very dissatisfied" and 10 means "Very satisfied", how do you feel about your life as a whole right now? | N/A | N/A |
| Health Utility Index (HUI) | Are you usually free of pain or discomfort?  How would you describe your usual ability to remember things?  How often do you use a wheelchair?  Are you able to walk at all? | Yes, for some questions | Abdominal Cramps, Body and Muscle Ache, Breast Pain, Chest Tightness or Pain, Headache, Lower Back Pain, Pelvic Pain, Sore Throat, Wheelchair Use, Audiogram, Environmental Sound Levels, Headphone Audio Levels, Noise Notifications, Headphone Notifications, Walking Speed, Step Length, Six-Minute Walk, Stair Speed: Up, Stair Speed: Down, Six-Minute Walk, Cardio Fitness, Walking Steadiness, Walking Asymmetry, Memory Lapse |
| Chronic conditions (CCC) | Have you had any asthma symptoms or asthma attacks in the past 12 months?  Do you have high blood pressure?  Do you currently take insulin for your diabetes?  In the past month, did you take pills to control your blood sugar? | Yes, for some questions | Inhaler Usage, Blood Pressure, Insulin Delivery, Blood Glucose |
| Vision (VIS) | Have you ever had glaucoma? | N/A | N/A |
| Sleep Apnea | Without the use of sleeping aids, how often do you usually have trouble going to sleep or staying asleep?  Using a scale from 0 to 10, where 0 means "no sleepiness" and 10 means "extremely sleepy", how would you assess your sleepiness during a typical day? | Yes, to some questions | Sleep Time In Bed, Sleep Time Asleep, Sleep Changes |
| Pregnancy (PRS) | Are you pregnant? | Yes | Pregnancy, Pregnancy Test, Progesterone Test |
| Menopause (MEN) | Have you had a menstrual period in the last 12 months? | Yes, to some questions | Mensturation |
| Fracture History (FRH) | Have you fallen in the past 12 months? | Yes, to some questions | Number of Times Fallen |
| Fracture Details (FRD) | Which bone(s) did you break or fracture (on that occasion)? | N/A | N/A |
| Medication Use (MEU) | Have you taken or used any other prescription medications in the past month? | N/A | N/A |
| Steroids and Osteoporosis Medications (SOM) | Have you ever used steroids administered by inhalation, for example, Flovent, Pulmicort or Vanceril? Do not include nasal sprays. | N/A | N/A |
| Height and Weight (HWT) | How tall are you without shoes on? | Yes | Height, Weight |
| Meat Consumption (MFC) | Now I'd like to ask about the use of omega-3 enriched eggs in the eggs and egg dishes you just reported. | N/A | N/A |
| Milk and Dairy Product Consumption (MDC) | hat kind of enriched milk substitutes do you usually drink or use on cereal? | N/A | N/A |
| Grain, Fruit and Vegetable Consumption (GFV) | Now, a few questions about grains, fruits and vegetables. Remember, think about all the foods you eat, both meals and snacks, at home and away from home. | N/A | N/A |
| Dietary Fat Consumption (DFC) | Remember, think about all the foods you eat, both meals and snacks, at home and away from home. | N/A | N/A |
| Water and Soft Drink Consumption (WSD) | How much water, in cups, do you usually drink at home? | N/A | N/A |
| Salt Consumption (SLT) | What type of salt is usually used? | N/A | N/A |
| Physical activities - Adults (PAA) | In the last seven days, how much time in total did you spend doing vigorous activities that caused you to be out of breath? | Yes | Steps, Walking + Running Distance, Active Energy, Exercise Minutes, Resting Energy, Stand Hour, Cardio Fitness, Workouts, Cycling Distance, Downhill Snow Sports Distance, NikeFuel, Pushes, Swimming Distance, Swimming Strokes, Weelchair Distance, Stand Minutes, Move Minutes |
| Physical activities for youth (PAY) | You have reported a total of ^DV_PAYTOTAL minutes of physical activity. Of these activities, were there any of vigorous intensity, meaning they caused you to be out of breath? | Yes, for most questions excluding questions which include location (e.g., In the last seven days, did you use active ways like walking or cycling to get to places such as [school, the bus stop, the shopping centre, work/school, the bus stop, the shopping centre/the bus stop, the shopping centre, work/the bus stop, the shopping centre] or to visit friends?) | Steps, Walking + Running Distance, Active Energy, Exercise Minutes, Resting Energy, Stand Hour, Cardio Fitness, Workouts, Cycling Distance, Downhill Snow Sports Distance, NikeFuel, Pushes, Swimming Distance, Swimming Strokes, Weelchair Distance, Stand Minutes, Move Minutes |
| Physical Activity of Children (CPA) | Over a typical or usual week, on how many days are you physically active for a total of at least 60 minutes per day? | Yes, for most questions excluding questions which include location (e.g., About how many hours a week do you usually take part in physical activity that makes you out of breath or warmer than usual: in your class time at school?). | Steps, Walking + Running Distance, Active Energy, Exercise Minutes, Resting Energy, Stand Hour, Cardio Fitness, Workouts, Cycling Distance, Downhill Snow Sports Distance, NikeFuel, Pushes, Swimming Distance, Swimming Strokes, Weelchair Distance, Stand Minutes, Move Minutes |
| Time Spent Outdoors (TSD) | During a weekday, did you go to school (including kindergarten)? | N/A | N/A |
| Sedentary Activities (SAC) | In the last seven days, how much of your free time did you spend: reading books, magazines or newspapers, including in electronic formats? Include time spent reading as part of your homework, but do not include time spent reading at work, during class time, while travelling in a vehicle or while exercising. | N/A | N/A |
| Neighbourhood Environment (NBE) | What is the main type of housing in your neighbourhood? | N/A | N/A |
| Smoking (SMK) | In your lifetime, have you smoked a total of 100 or more cigarettes (about 4 packs)? | N/A | N/A |
| Electronic Cigarette (ELC) | Have you ever tried an electronic cigarette, also known as an e-cigarette? | N/A | N/A |
| Exposure to Second-Hand Smoke (ETS) | Is smoking allowed inside this home? | N/A | N/A |
| Exposure to Second-Hand Vapor (ETV) | Overall, in the past month, how often were you exposed to second-hand vapour inside this home? | N/A | N/A |
| Alcohol Use (ALC) | During the past 12 months, that is have you had a drink of beer, wine, liquor or any other alcoholic beverage? | N/A | N/A |
| Illicit Drug Use (IDU) | Have you ever used or tried marijuana, cannabis or hashish? | N/A | N/A |
| Sexual Behaviour (SXB) | In the past 12 months, have you had sexual intercourse? | Yes, to some questions | Sexual Activity |
| Birth Control (BCL) | In total, over your lifetime, how many years did you use birth control pills? | Yes | Contraceptives |
| Maternal Breastfeeding (MBF) | Have you ever given birth? | Yes, to some questions | Pregnancy |
| Breastfeeding (BRF) | Did you breastfeed your baby? | N/A | N/A |
| Pregnancy Information (PRG) | Did [you/she] smoke during [your/her] pregnancy? | N/A | N/A |
| Birth Information (BIR) | How much did you weigh at birth? | N/A | N/A |
| Breastfeeding Information (BRI) | For how long did [you/she] breastfeed? | N/A | N/A |
| Labour market activity minimum - LMAM | Last week, did you work at a job or business? (regardless of the number of hours) | N/A | N/A |
| Labour market activity Sublock Labour force status - LMA2 | Last week, did you have a job to start at a definite date in the future? | N/A | N/A |
| Labour market activity Sublock Class of worker - LMA3 | Were you an employee or self-employed? | N/A | N/A |
| Industry (LMA4) | What was the name of your business? | N/A | N/A |
| Labour market activity Sublock Occupation - LMA5 | What was your work or occupation? | N/A | N/A |
| Labour Market Hours of Work (LMH) | On average, how many hours do you usually work per week? | N/A | N/A |
| Immigration Block (IMG) | In what country were you born? | N/A | N/A |
| Aboriginal minimum - AMB | Are you an Aboriginal person, that is, First Nations, Métis or Inuk/Inuit? First Nations includes Status and Non-Status Indians. | N/A | N/A |
| Population Group (PG) | You may belong to one or more racial or cultural groups on the following list. | N/A | N/A |
| Language Extended (LAE) | Of English or French, which language(s) do you speak well enough to conduct a conversation? | N/A | N/A |
| Education Minimum Block with Concept (EDM) | What type of educational institution [are you attending/did you attend]? | N/A | N/A |
| Education Sublock School attendance "currently" - ESC1 | Are you currently attending school, college, CEGEP or university? | N/A | N/A |
| Total Personal Income (TPI) | Can you estimate in which of the following groups your personal income falls? | N/A | N/A |
| Total Household Income (THI) | Can you estimate in which of the following groups your household income falls? | N/A | N/A |
| Administration Information (ADM) | Was this interview conducted on the telephone or in person? | N/A | N/A |
| **Activity Monitor** | Use of activity monitor for a week | Yes | Steps, Walking + Running Distance, Active Energy, Exercise Minutes, Resting Energy, Stand Hour, Cardio Fitness, Workouts, Cycling Distance, Downhill Snow Sports Distance, NikeFuel, Pushes, Swimming Distance, Swimming Strokes, Wheelchair Distance, Stand Minutes, Move Minutes |

Table 3: CCHS Measures related to Possible AH Variables

| **Type** | **Question Example** | **AH** | **Possible AH Variables** |
| --- | --- | --- | --- |
| Respondent Availability | May I speak to [First name of household contact] [Last name of household contact]? | Yes | The owner of the phone is the respondent |
| Proxy Respondent (PRX) | Is there someone [Minimum age of person providing proxy interview] or older who could provide us with some information on behalf [First name of household contact]? | N/A | N/A |
| Verification (VER2) | Are you [First name of specific respondent] [Last name of specific respondent]?  What is your date of birth? | Yes | Date of Birth, respondent is the owner of the phone |
| Date of birth (AGE) | What is [your] date of birth? | Yes | Date of Birth |
| Sex and Gender (GDRA) | What was [your] sex at birth? | Yes, for some questions | Biological Sex |
| Relationship with confirmation (RWC) | What is the relationship…of: [Name of specific respondent] ([Age of specific respondent]) to: [Name of secondary respondent] ([Age of secondary respondent])? | N/A | N/A |
| Main activity (MAC) | In the past 12 months, was your main activity working at a job or business? | N/A | N/A |
| Main activity (MA) | During the past 12 months, what was your main activity? | N/A | N/A |
| Main Activity (EDC) | Are you currently attending a school, college, CEGEP or university? | N/A | N/A |
| General health (GEN) | In general, how is your health?  Thinking about the amount of stress in your life, how would you describe most of your days? | N/A | N/A |
| Life satisfaction measures (LSM) | How do you feel aboutyour life as a whole right now? | N/A | N/A |
| Pregnancy | Are you pregnant? | Yes | Pregnancy, Pregnancy Test Result, Progesterone Test Result |
| Height and weight (HWT) | How tall are you without shoes on? | Yes | Height, Weight |
| Weight perception (WTP) | Do you consider yourself overweight, underweight or just about right? | N/A | N/A |
| COVID-19 (COV2) | In the last 3 months, have you experienced any of the symptoms that led you to believe that you had COVID-19, such as fever, headache, sore throat, runny nose, difficulty breathing or tiredness?  Have you been vaccinated against COVID-19? | Yes, for some questions | Headache, Fever, Runny Nose, Sore Throat, Shortness of Breath, Wheezing, Immunizations |
| Vaccination passeport COVID-19 (PVC) | Some public health authorities are considering establishing a COVID-19 vaccination passport or have already done so.  Is such a passport a motivation for you to get vaccinated? | N/A | N/A |
| COVID-19 (COV3) | If an additional dose of the COVID-19 vaccine is offered to stimulate your immune system or to fight against variants, how likely is it that you would get it? | N/A | N/A |
| Chronic conditions (CCC) | Do you currently take insulin for your diabetes? Do you have high blood pressure?  Do you currently take insulin for your diabetes?  In the past month, did you take pills to control your blood sugar?  Do you have heart disease? | Yes, for some questions | Inhaler Usage, Blood Pressure, Insulin Delivery, Blood Glucose, Medication That Affects Heart, Dietary Cholesterol, Mood Changes, High/Low Heart Rate Notifications, Irregular Rhythm Notifications |
| Chronic conditions (CC1) | Do you have an anxiety disorder?  Do you have Alzheimer's disease or any other dementia? | Yes, for some questions | Mood Changes, Memory Lapse, Fatigue |
| Abilities (WDM) | Do you have difficulty doing any of these activities? Difficulty hearing, even if using a hearing aid Do you have difficulty doing any of these activities?  Difficulty walking or climbing steps | Yes, for some questions | Audiogram, Environmental Sound Levels, Headphone Audio Levels, Noise Notifications, Headphone Notifications, Walking Speed, Step Length, Six-Minute Walk, Stair Speed: Up, Stair Speed: Down, Six-Minute Walk, Cardio Fitness, Walking Steadiness, Walking Asymmetry |
| Injuries (INJ) | In the past 12 months, did you have any of the following injuries? A head injury or concussion | N/A | N/A |
| Oral health (OHM) | In general, how would you rate the health of your mouth | N/A | N/A |
| Oral health (OHM3) | How often do you usually see a dental professional? | N/A | N/A |
| Changes made to improve health (CIH) | In the past 12 months, did you do anything to improve your health? (For example, lost weight, quit smoking, increased exercise.) | Yes, for some questions | Weight, Steps, Walking + Running Distance, Workouts, Cycling Distance |
| Eating Habits (EAH) | In the past 30 days, how many times did you eat food from a restaurant?  In the past 30 days, how many times did you eat the following fruits and vegetables? | N/A | N/A |
| Physical activities - adults 18 years and older (PAA) | In the past 7 days, did you do sports, fitness or recreational physical activities?  In the past 7 days, on which days did you do these other activities that made you sweat at least a little and breathe harder? | Yes, for the majority of questions excluding questions which include location (e.g., In the past 7 days, did you do any other physical activities while at work, in or around your home or while volunteering?) | Steps, Walking + Running Distance, Active Energy, Exercise Minutes, Resting Energy, Stand Hour, Cardio Fitness, Workouts, Cycling Distance, Downhill Snow Sports Distance, NikeFuel, Pushes, Swimming Distance, Swimming Strokes, Weelchair Distance, Stand Minutes, Move Minutes |
| Physical activities for youth (PAY) | In the past 7 days, did you do any other physical activities?  You have reported a total of [total hours of active transportation + total hours of recreational physical activities + total hours of other physical activity] hours of physical activity. Of these activities, were there any of vigorous intensity, meaning they caused you to be out of breath? | Yes, for most questions excluding questions which include location (e.g., In the past 7 days, did you do sports, fitness, or recreational physical activities while at [school or day camp, including during physical education classes, during your breaks and any other time you played indoors or outdoors/school, including during physical education classes, during your breaks and any other time you played indoors or outdoors/day camp, including any time you played indoors or outdoors]?) | Steps, Walking + Running Distance, Active Energy, Exercise Minutes, Resting Energy, Stand Hour, Cardio Fitness, Workouts, Cycling Distance, Downhill Snow Sports Distance, NikeFuel, Pushes, Swimming Distance, Swimming Strokes, Wheelchair Distance, Stand Minutes, Move Minutes |
| Use of protective equipment (UPE) | In the past 12 months, have you participated in any of these activities?  1: Bicycling  2: In-line skating or rollerblading  3: Downhill skiing  4: Snowboarding  5: Skateboarding  6: Playing ice hockey | Yes, to questions identifying activities (however AH does not have information on protective equipment use) | Workouts, Cycling Distance, Downhill Snow Sports Distance, Swimming Distance |
| Sedentary behaviours (SBE) | On a school or work day, how much of your free time did you spend watching television or a screen on any electronic device while sitting or lying down? | N/A (while in theory sedentary behaviour could be measured from smartphones, the questions in CCHS ask how much free time respondents spent watching television or a screen, which is not something AH can capture) | N/A |
| Sleep (SLP) | How long do you usually spend sleeping each night?  How often do you have trouble going to sleep or staying asleep? | Yes, for some questions | Sleep Time In Bed, Sleep Time Asleep, Sleep Changes |
| Current smoking status (CSS) | Have you ever smoked a whole cigarette? | N/A | N/A |
| Smoking – past use (SPU) | Have you ever smoked cigarettes daily? | N/A | N/A |
| Electronic cigarettes and vaping (ECV) | Have you ever tried an e-cigarette or vaping device? | N/A | N/A |
| Electronic cigarettes and vaping 2 (ECV2) | During the past 30 days, on how many days did you vape the following products? An e-liquid with nicotine | N/A | N/A |
| Alcohol use (ALC) | Have you ever had a drink in your lifetime? | N/A | N/A |
| Medication use — pain relievers (PRM) | In the past 12 months, have you taken any codeine products? | N/A (AH may not be capable of tracking medication intake but it does allow for pain symptom self-reporting) | N/A |
| Cannabis use (CAN) | Have you ever used or tried cannabis? | N/A | N/A |
| Maternal experiences (MEX) | Are you taking a vitamin supplement containing folic acid?  Have you given birth in the past 5 years? | Yes, for some questions | Folate, Iron, Weight, Pregnancy, Pregnancy Test, Progesterone Test, Lactation |
| Smoking during maternal experience (MXS) | In the 3 months before your pregnancy with [your last child], or before you realized you were pregnant, did you smoke cigarettes? | N/A | N/A |
| Alcohol use during maternal experience (MXA) | In the 3 months before your pregnancy with [your last child], or before you realized you were pregnant, did you drink any alcohol? | N/A | N/A |
| Flu shots (FLU) | In the past 12 months, have you had a seasonal flu vaccine? | N/A | N/A |
| Regular health care provider (RHC) | Which of the following health care providers do you regularly consult with? | N/A | N/A |
| Labour market activities (LMAM) | Last week, did you work at a job or business? | N/A | N/A |
| Labour market activities (LMAM3) | Were you an employee or self-employed? | N/A | N/A |
| Labour market activities (LMAM4) | What kind of business, industry or service was this? | N/A | N/A |
| Labour market activities (LMAM5) | What kind of work were you doing? | N/A | N/A |
| Labour market activities (LMAM6) | [Excluding overtime, on average, how many paid hours do you usually work per week?/On average, how many hours do you usually work per week?] | N/A | N/A |
| Labour market activities (LBF) | Did you have more than one job or business last week? | N/A | N/A |
| Telework (LM) | In the past 30 days, in which of these locations did you work the most hours? | N/A | N/A |
| Place of birth, immigration and citizenship (IM) | Where were you born? | N/A | N/A |
| Indigenous identity (ABM) | Are you First Nations, Métis or Inuk (Inuit)? | N/A | N/A |
| Population group (PG) | Are you? [List of Population Groups] | N/A | N/A |
| Language | Can you speak English or French well enough to conduct a conversation? | N/A | N/A |
| Sexual orientation (SOR) | What is your sexual orientation? | N/A | N/A |
| Home care services (HMC) | In the past 12 months, what type of home care services have been received? | N/A | N/A |
| Insurance coverage (INL) | Do you have insurance that covers all or part of the cost of your long term care, including home care? | N/A | N/A |
| Insurance coverage (INP) | Do you have insurance that covers all or part of the cost of your prescription medications? | N/A | N/A |
| Prescriptions cost (PCN) | In the past 12 months, did [you] do any of the following because of the cost of [your] prescriptions? | N/A | N/A |
| Food security (FSC) | The food that you [and other household members] bought just didn't last, and there wasn't any money to get more. | N/A | N/A |
| Administration information (ADMC) | For which province or territory is your health number? | N/A | N/A |
| Total household income (INC) | What is your best estimate of total household income received by all household members, from all sources, before taxes and deductions, during the year ending December 31, [Past year]? | N/A | N/A |

Table 4: PASS (Adult) Measures related to Possible AH Variables

| **Type** | **Description** | **Source** | **AH** | **Possible AH Variables** |
| --- | --- | --- | --- | --- |
| Physical activity guideline adherence | Percentage of adults who meet physical activity guidelines by accumulating at least 150 minutes of moderate-to-vigorous physical activity each week, in bouts of 10 minutes or more | CHMS (2016-2017) | Yes | Steps, Walking + Running Distance, Active Energy, Exercise Minutes, Resting Energy, Stand Hour, Cardio Fitness, Max, Workouts, Cycling Distance, Downhill Snow Sports Distance, NikeFuel, Pushes, Swimming Distance, Swimming Strokes, Wheelchair Distance, Stand Minutes, Move Minutes |
| Total moderate-to-vigorous physical activity amount | Average number of minutes per day adults are engaged in moderate-to-vigorous physical activity | CHMS (2016-2017) | Yes | Steps, Walking + Running Distance, Active Energy, Exercise Minutes, Resting Energy, Stand Hour, Cardio Fitness, Max, Workouts, Cycling Distance, Downhill Snow Sports Distance, NikeFuel, Pushes, Swimming Distance, Swimming Strokes, Wheelchair Distance, Stand Minutes, Move Minutes |
| Occupational physical activity and active chores amount | Average number of hours per week adults report doing physical activities while at work, in or around their home or while volunteering | CCHS (2018) | N/A | N/A |
| Leisure time physical activity amount | Average number of hours per week adults report doing sports, fitness or recreational physical activities, organized or non-organized, that lasted a minimum of 10 continuous minutes | CCHS (2018) | Yes | Steps, Walking + Running Distance, Active Energy, Exercise Minutes, Resting Energy, Stand Hour, Cardio Fitness, Max, Workouts, Cycling Distance, Downhill Snow Sports Distance, NikeFuel, Pushes, Swimming Distance, Swimming Strokes, Wheelchair Distance, Stand Minutes, Move Minutes |
| Sports participation amount | Percentage (%) of population who reported regularly participating in any sports during the past 12 months | General Social Survey (GSS) (2016) | Yes | Workouts, Cycling Distance, Downhill Snow Sports Distance, NikeFuel, Pushes, Swimming Distance, Swimming Strokes |
| Active travel amount | Percentage (%) of adults who report walking or cycling to work or school/Average number of hours per week adults report using active ways like walking or cycling to get to places | CCHS (2018) | N/A | N/A |
| Intention level | Percentage (%) of adults who, when thinking about the next six months, intend to be physically active | Physical Activity Monitor (PAM)(2014-2015) | N/A | N/A |
| Enjoyment level | Percentage (%) of adults who report that physical activity is generally pleasant | PAM (2014-2015) | N/A |  |
| Confidence level | Percentage (%) of adults who report they are confident that they could regularly do a total of 30 minutes or more of moderate physical activity three or four times a week | PAM (2014-2015) | N/A |  |
| Physical health status | Percentage (%) of adults who report their health is "very good" or "excellent" | CCHS (2019) | N/A |  |
| Mental health status | Percentage (%) of adults who report their mental health is "very good" or "excellent" | CCHS (2019) | N/A |  |
| Presence of parks and recreation facilities | Percentage (%) of adults that "somewhat agree" or "strongly agree" that their neighbourhood has several free or low cost recreation facilities, such as parks, walking trails, bike paths, recreation centers, playgrounds, public swimming pools, etc. | CCHS RR (2011) | N/A |  |
| Presence of active transport infrastructure | Percentage (%) of adults who report their community has infrastructure that supports walking or biking (well-maintained sidewalks or designated bike areas for biking) | CCHS RR (2011) | N/A |  |
| Shower access at work | Percentage (%) of adults who report having access to showers or change rooms at or near work | CCHS (2007-2008) | N/A | N/A |
| Total sedentary time amount | Average number of hours per day adults spend sedentary, excluding sleep time | CHMS (2016-2017) | Yes | Steps, Walking + Running Distance, Active Energy, Exercise Minutes, Resting Energy, Stand Hour, Cardio Fitness, Max, Workouts, Cycling Distance, Downhill Snow Sports Distance, NikeFuel, Pushes, Swimming Distance, Swimming Strokes, Wheelchair Distance, Stand Minutes, Move Minutes |
| Recreational screen time amount | Average number of hours per day adults report watching television, DVDs, or videos or spending time on a computer, tablet, or other hand-held electronic device e.g. watching videos, playing computer/video games, emailing or surfing the Interne | CHMS (2014-2015) | N/A | N/A |
| Nighttime sleep amount | Average number of hours adults report sleeping in a 24-hour period | CHMS (2014-2015) | Yes | Sleep Time In Bed, Sleep Time Asleep |
| Sleep quality — sleep continuity | Percentage (%) of adults who report having trouble going to sleep or staying asleep "most of the time" or "all of the time" | CHMS (2014-2015) | Yes | Sleep Time In Bed, Sleep Time Asleep , Sleep Changes |

Table 5: PASS (Children and Youth) Measures related to Possible AH Variables

| **Type** | **Description** | **Source** | **AH** | **Possible AH Variables** |
| --- | --- | --- | --- | --- |
| Physical activity guideline adherence | Percentage (%) of children and youth who meet physical activity recommendations by accumulating at least 60 minutes of moderate-to-vigorous physical activity per day | CHMS (2016-2017) | Yes | Steps, Walking + Running Distance, Active Energy, Exercise Minutes, Resting Energy, Stand Hour, Cardio Fitness, Max, Workouts, Cycling Distance, Downhill Snow Sports Distance, NikeFuel, Pushes, Swimming Distance, Swimming Strokes, Wheelchair Distance, Stand Minutes, Move Minutes |
| Total moderate-to-vigorous physical activity amount | Average number of minutes per day children and youth are engaged in moderate-to-vigorous physical activity | CHMS (2016-2017) | Yes | Steps, Walking + Running Distance, Active Energy, Exercise Minutes, Resting Energy, Stand Hour, Cardio Fitness, Max, Workouts, Cycling Distance, Downhill Snow Sports Distance, NikeFuel, Pushes, Swimming Distance, Swimming Strokes, Wheelchair Distance, Stand Minutes, Move Minutes |
| 24-hour movement | Percentage (%) of children and youth who meet the Canadian 24-Hour Movement Guidelines for Children and Youth | CHMS (2014-2015) | Yes | Steps, Walking + Running Distance, Active Energy, Exercise Minutes, Resting Energy, Stand Hour, Cardio Fitness, Max, Workouts, Cycling Distance, Downhill Snow Sports Distance, NikeFuel, Pushes, Swimming Distance, Swimming Strokes, Wheelchair Distance, Stand Minutes, Move Minutes |
| School physical activity amount | Average number of hours per week youth in Grades 6 to 10 report taking part in physical activity that makes them out of breath or warmer than usual during class time at school/Average number of hours per week that parents report their children spend doing physical activity during class time at school | Health Behaviours in School-aged Children  (HBSC) (2014) and CHMS (2018-2019) | N/A | N/A |
| Sports participation amount (leisure time) | Percentage (%) of Canadian parents who report that their children participated in sports in the last 12 months | Canadian Health Survey on Children and Youth (CHSCY) (2019) | Yes (although in this case it wouldn’t be reported by the parents) | Workouts, Cycling Distance, Downhill Snow Sports Distance, NikeFuel, Pushes, Swimming Distance, Swimming Strokes |
| Active play amount (leisure time) | Percentage (%) of children who accumulate 3 hours or less per week of active play (unstructured physical activity) outside of school | CHMS (2016-2017) | N/A | N/A |
| Active travel amount | Percentage (%) of youth who report walking or cycling to work or school/Average number of hours per week youth report using active ways like walking or cycling to get to placesschool/Average number of hours per week adults report using active ways like walking or cycling to get to places | CCHS (2018) | N/A | N/A |
| Physical health status | Percentage (%) of youth who report their health is "very good" or "excellent"/Percentage (%) of parents who report the health of their child is "very good" or "excellent" | CCHS (2019), CHMS (2018-2019) | N/A | N/A |
| Mental health status | Percentage (%) of youth who report their mental health is "very good" or "excellent" | CCHS (2019) | N/A | N/A |
| Level of parental support | Percentage (%) of Canadian parents who report "often" or "very often" playing active games with their children in the past year | PAM (2014-2015) | N/A | N/A |
| Level of peer support | Percentage (%) of youth in Grades 9 and 10 who report that most of their friends "often" participate in organized sports activities with others | HBSC (2014) | N/A | N/A |
| Level of community safety | Percentage (%) of Canadian parents who identify safety concerns as a barrier to their children’s physical activity | CHSCY (2019) | N/A | N/A |
| Presence of parks and recreation facilities | Percentage (%) of youth who "somewhat agree" or "strongly agree" that their neighbourhood has several free or low cost recreation facilities, such as parks, walking trails, bike paths, recreation centers, playgrounds, public swimming pools, etc. | CCHS RR (2011) | N/A |  |
| Supportive policies at school | Percentage (%) of schools that have a committee that overseas policies and practices concerning physical activity (e.g. health action team) | HBSC — Admin (2014) | N/A |  |
| Sedentary behaviour recommendation adherence | Percentage (%) of children and youth who report meeting sedentary behaviour recommendations by spending 2 hours or less per day watching television or using a computer during leisure-time | CHMS (2018-2019) | N/A |  |
| Amount of sedentary time | Average number of hours per day children and youth spend sedentary, excluding sleep time | CHMS (2016-2017) | Yes | Steps, Walking + Running Distance, Active Energy, Exercise Minutes, Resting Energy, Stand Hour, Cardio Fitness, Max, Workouts, Cycling Distance, Downhill Snow Sports Distance, NikeFuel, Pushes, Swimming Distance, Swimming Strokes, Wheelchair Distance, Stand Minutes, Move Minutes |
| Recreational screen time amount | Average number of hours per day youth report watching television, DVDs, or videos or spending time on a computer, tablet, or other hand-held electronic device e.g. watching videos, playing computer/video games, emailing or surfing the Internet | CHMS (2018-2019) | N/A | N/A |
| Time spent outdoors | Average number of hours per day children spend outside | CHMS (2014-2015) | N/A | N/A |
| Sleep recommendation adherence | Percentage (%) of children and youth who report meeting sleep recommendations by obtaining adequate sleep: 9-11 hours per night for ages 5 to 13 years and 8-10 hours per night for ages 14 to 17 years | CHMS (2014-2015) | Yes | Sleep Time In Bed, Sleep Time Asleep |
| Amount of sleep in 24-hour period | Average number of hours children and youth report sleeping in a 24-hour period | CHMS (2014-2015) | Yes | In Bed, Asleep |
| Sleep quality — sleep continuity | Percentage (%) of children and youth who report having trouble going to sleep or staying asleep "most of the time" or "all of the time" | CHMS (2014-2015) | Yes | In Bed, Asleep, Sleep Changes |
| Electronic media in the bedroom | Percentage (%) of children and youth who use electronic devices in the bedroom before falling asleep | CHSCY (2019) | N/A (although this information can be obtained from devices, it is not from AH) | N/A |

Table 6: Characteristics of Apple, Fitbit and Samsung Users

| Device | % Market Share | % of Users with a Bachelor’s degree and Above | % of Users in Larger Cities | % Gender | % of Users with High Income | Predominant Age (18-64) |
| --- | --- | --- | --- | --- | --- | --- |
| Apple | 41% | 57% | 68% | Equal share of male and female users | 50% High Income | 18-29 years (35%) |
| Fitbit | 38% | 46% | 61% | 61% Female | 42% High Income | 50-64 (32%) |
| Samsung | 13% | 50% | 60% | 57% Male | 47% High Income | 18-29 (34%) |

Table 7: Canadian provincial health laws deemed substantially similar to PIPEDA

| **Province** | **Provincial privacy laws** |
| --- | --- |
| Ontario | Personal Health Information Protection Act (PHIPA) |
| New Brunswick | Personal Health Information Privacy and Access Act (PHIPAA) |
| Newfoundland and Labrador | Personal Health Information Act (PHIA) |
| Nova Scotia | Personal Health Information Act (PHIA) |

Table 8: 10 principles of PIPEDA

| **Principle** | **Description** |
| --- | --- |
| Accountability | PII is the responsibility of the organization that controls it. The organization must designate individuals that are accountable for compliance with PIPEDA’s principles. |
| Identifying Purposes | The purposes for the collection of PII must be stated by the organization before or during collection. |
| Consent | Consent of individuals whose data is being collected, used or disclosed is required. |
| Limiting Collection | Collection of PII will be limited to what is necessary to fulfill the purposes outlined by the organization. The information must be collected legally. |
| Limiting Use, Disclosure, and Retention | Use and disclosure of PII will be limited to the purposes for which it was collected, except when required by law or if consent from the individual is sought again. The information will be retained until the purposes are fulfilled. |
| Accuracy | PII will be as accurate, up-to-date and complete as required to complete the purposes outlined for its collection. |
| *Safeguards* | *Appropriate safeguards to the sensitivity of the information will be employed by the organization to protect PII.* |
| Openness | Policies and practices concerning the management of PII will be made available. |
| Individual Access | If it is requested by an individual, organizations must inform individuals of the existence of PII and related uses. An individual can contest the accuracy of the information and have it changed. |
| Challenging Compliance | Individuals can challenge compliance with the above principles by an organization. |

Table 9 : Examples of physical, administrative and technical safeguards recommended by IPC

| Physical | Locked cabinets |
| --- | --- |
|  | Restricted office access |
|  | Alarm systems |
| Administrative | Security clearance |
|  | Confidentiality agreements |
|  | Training |
|  | Regular monitoring of compliance with security policies |
| Technical | Login and password |
|  | Encryption |
|  | Firewalls |
|  | Malware detection software |
